# Diagnostic assessment of foetal brain malformations with intra-uterine MRI versus perinatal post-mortem MRI

**DOI:** 10.1007/s00234-019-02218-9

**Published:** 2019-05-10

**Authors:** Stacy K. Goergen, Ekaterina Alibrahim, Nishentha Govender, Alexandra Stanislavsky, Christian Abel, Stacey Prystupa, Jacquelene Collett, Susan C. Shelmerdine, Owen J. Arthurs

**Affiliations:** 1 Monash Imaging, Clayton, Victoria Australia; 2 School of Clinical Sciences, Monash University, Clayton, Victoria Australia; 3 Department of Medical Imaging, Royal Women’s Hospital, Parkville, Victoria Australia; 4 Department of Medical Imaging, John Hunter Hospital, Newcastle, New South Wales Australia; 5 Department of Anatomical Pathology, Monash Health, Clayton, Victoria Australia; 6 Department of Anatomical Pathology, Royal Children’s Hospital, Parkville, Victoria Australia; 7 Department of Radiology, Great Ormond Street Hospital for Children NHS Foundation Trust, London, WC1N 3JH UK; 8 UCL Great Ormond Street Institute of Child Health, London, UK

**Keywords:** Fetus, Autopsy, Magnetic resonance imaging, Brain, Congenital, Termination of pregnancy

## Abstract

**Purpose:**

To evaluate differences in diagnostic yield of intra-uterine foetal (iuMR) and post-mortem MRI (PMMR) for complex brain malformations, using autopsy as the reference standard.

**Methods:**

In this retrospective, multicentre study spanning 2 years, we reviewed 13 terminated singleton pregnancies with a prenatal ultrasound finding of complex foetal cerebral abnormalities, referred for both iuMR and PMMR. The iuMR and PMMR studies of the brain were reported independently by two groups of radiologists, blinded to each other’s reports. Descriptive statistics were used to compare differences in intracranial abnormalities with autopsy (and genetic testing, where present) as reference standard.

**Results:**

The median gestational age at termination was 24.6 weeks (IQR 22–29) with median time between delivery and PMMR of 133 h (IQR 101–165). There was full concordance between iuMR and PMMR findings and autopsy in 2/13 (15.3%) cases. Partial concordance between both imaging modalities was present in 6/13 (46.2%) and total discordance in the remainder (5/13, 38.5%). When compared to autopsy, PMMR missed important key findings specifically for neuronal migration and cerebellar anomalies, whereas iuMR appeared to overcall CSF space abnormalities which were less crucial to reaching the final overall diagnosis.

**Conclusions:**

iuMR should be performed to improve foetal phenotyping where there is a prenatal ultrasound for complex foetal brain abnormalities. Reliance on PMMR alone is likely to result in misdiagnosis in a majority of cases.

**Electronic supplementary material:**

The online version of this article (10.1007/s00234-019-02218-9) contains supplementary material, which is available to authorized users.

## Introduction

Foetal brain malformations occur in 2–3 per 1000 pregnancies [1–3] and are amongst the commonest reasons for terminations of pregnancy [4, 5] given their poor long-term outcomes [6–8]. A genetic basis is frequently responsible [9–13], and there is a risk of disease recurrence in future pregnancies depending upon the nature of the abnormality [14]. An accurate prenatal diagnosis is therefore of utmost importance, for targeting subsequent genetic testing of the fetus and for parental counselling regarding recurrence risk.

Prenatal ultrasound is the primary imaging modality for detecting congenital anomalies, with a high concordance rate for brain abnormalities of approximately 75–80% [15–17] (when compared with post-natal imaging and/or autopsy). Recent technological advances, specifically in ultrafast MRI sequences, mean that intra-uterine foetal MRI (iuMR) is becoming more widely available. It is reported to improve upon the sonographic accuracy for foetal brain anomalies by an additional 16% [18, 19] and help in refining prognostic information for 20% of cases [20–22]. Nevertheless, iuMR is not widely available even in tertiary obstetric centres and some women find undertaking the examination physically challenging [23, 24]. Both clinician and patient may also perceive the additional information as unnecessary, if there is a high confidence in the sonographic diagnosis and a post-mortem examination following termination of pregnancy has been agreed to confirm the diagnosis.

Post-mortem diagnoses of foetal brain malformations have traditionally been provided by brain autopsy, although declining rates of parental consent [25–32] for this invasive procedure have led to perinatal post-mortem MRI (PMMR) becoming an increasingly utilised adjunct, and sometimes replacement, tool. Large cohort studies have shown a high concordance rate of 74–92% with brain autopsy [31, 33], and PMMR can also provide additional information over autopsy, especially where there is marked intracranial maceration [31]. In some centres, the brain is no longer examined when PMMR is normal.

It could be therefore argued that given the high concordance rates of both iuMR and PMMR with formal autopsy, only one of these imaging studies is necessary. A recent work by Izzo et al. [34] has challenged this perception. Using the combined results from iuMR and PMMR in the same 53 foetuses as a reference standard, they found that iuMR detected 79% of brain abnormalities but only 45% were detected by PMMR [34], suggesting some loss of information if only performing PMMR. The true diagnostic accuracy rate in their study remains unknown, as they lacked autopsy data in their cohort.

The aim of our study was to evaluate differences in diagnostic yield of foetal brain malformations for iuMR and PMMR, using autopsy as the reference standard. We intended to determine whether one or both are required for accurate diagnoses of foetal brain malformations when malformation was suspected based on prenatal US.

## Materials and methods

### Ethical approval and consent

A site-specific waiver of formal ethics approval was obtained from three different participating institutions to allow for retrospective data collection and sharing of anonymised clinical and imaging information. All women taking part provided verbal consent for iuMR studies and written informed consent was obtained from all parents for PMMR and autopsy, where performed.

### Study cohort

A retrospective review of the radiology information systems (RIS) of three different institutions was conducted for a 2-year period spanning 1 January 2015–1 January 2017. Each of the three institutions had capabilities of performing both iuMR and PMMR examinations on site.

Cases were reviewed based on the following inclusion criteria:

1. Tertiary prenatal ultrasound study suggesting a foetal brain malformation (at ≥ 19 weeks gestation)
2. Subsequent iuMR showing a brain abnormality (not necessarily the same abnormality)
3. Termination of pregnancy for foetal brain abnormality by parental request
4. Perinatal PMMR

No formal exclusion criteria were set. Demographic details obtained for each patient included date of birth/death, gestational age at termination of pregnancy, prenatal history, method used to terminate pregnancy and prenatal sonographic diagnosis (thus the referral indications for iuMR and PMMR examinations). The time between termination of pregnancy and PMMR was calculated in hours, taking the time of foetal demise as either time of foetal intracardiac injection, time of maternal administration of oral mifepristone or time of foetal demise during delivery. The results of a conventional post-mortem examination by a foetal pathologist at the institution of origin were collated, where this was available.

### Magnetic resonance imaging

iuMR and PMMR examinations were performed according to local institutional protocols on either a 3T Ingenia (Philips Healthcare, Amsterdam, The Netherlands), 3T GE Excite (GE Medical Systems, Milwaukee, USA) or 1.5T Avanto (Siemens, Erlangen, Germany) MRI scanner.

#### Intra-uterine fetal MRI

For all cases, the entire fetus was imaged in three orthogonal planes with T2-weighted single shot echoplanar imaging with the addition of T1 axial-, diffusion- and susceptibility-weighted imaging in the axial plane for the cranial portion of the study. The intracranial diagnoses were predominantly made by assessment of the T2-weighted imaging, and the imaging parameters for this sequence are given in Table 1.

**Table 1:** Intra-uterine MR parameters for axial T2-weighted acquisition of intracranial structures

	Site 1 3T Philips Ingenia (*N* = 9)	Site 2 3T GE Excite (*N* = 2)	Site 3 3T Siemens Magnetom Verio (*N* = 2)
Coil	32-channel body array	8-channel cardiac coil	6-channel body + spine array; 12 channels
T2 W seq	Ultrafast SE	SSFSE	HASTE
TR/TE (ms)	10,000/3000	4000/327	2000/103
FOV (mm)	180 (dS zoom)	260 (dep on patient)	340
Slice	3 mm	3 mm/skip 0.3	3 mm
Matrix	184 × 150	256 × 224	256 × 256
Voxel	1.0 × 1.1 × 3	1.0 × 1.1 × 3.3	1.3 × 1.3 × 3
Excitations	2	1	1
Bandwidth (Hz/pixel)	452.9	31.25	781

*seq* sequence, *TR* repetition time, *TE* echo time, *TI* inversion time, *ax* axial, *cor* coronal, *sag* sagittal, *FOV* field of view, *Hz* Hertz, *SE* spin echo, *SSFSE* single shot fast spin echo, *HASTE* half-Fourier acquisition single shot turbo spin echo

#### Perinatal post-mortem MRI

PMMR was performed within 4–5 days of delivery and in all cases. The entire fetus was imaged, with T2-weighted imaging in three orthogonal planes and T1, susceptibility-weighted (SWI) and diffusion-weighted imaging (DWI) in the axial plane only. Scan parameters for T2-weighted imaging performed at PMMR are provided in Table 2.

**Table 2:** Postmortem MR parameters for axial T2-weighted acquisition of the foetal head

	Site 1 1.5T Siemens Symphony (*N* = 8)	Site 1 3T Philips Ingenia (*N* = 1)	Site 2 3T GE Excite (*N* = 2)	Site 3 3T Siemens Magnetom Verio (*N* = 2)
Coil	16-channel head	8-channel paediatric head	12-channel knee or 16-channel head	12-channel head with 4-channel neck
T2 W seq (ax/cor/sag)	SPACE	TSE–ETL 30	FSE (ax/cor/sag)	TSE
TR/TE	3200/388 TI 1100	3500/160	3320/110	2500 / 73
FOV	250	100	160 (ax) 140 (sag/cor)	150
Slice (mm)	1	3 no skip	3/skip 0.3	3
Matrix	512 × 516	168 × 168	192 × 320 (ax) 192 × 352 (cor/sag)	163 × 256
Voxel (mm)	0.5 × 0.5 × 1.0	0.6 × 0.6 × 3	0.7 × 0.4 × 3.3	0.7 × 0.6 × 3
Excitations	1	1	2	1
Bandwidth (Hz/pixel)		435	31.25	222

*seq* sequence, *TR* repetition time, *TE* echo time, *TI* inversion time, *ax* axial, *cor* coronal, *sag* sagittal, *FOV* field of view, *Hz* Hertz, *TSE* turbo spin echo, *ETL* echo train length, *FSE* fast spin echo, *SPACE* sampling perfection with application optimised contrasts using different flip angle evolution

### Termination of pregnancy

Feticide by intracardiac injection was performed by qualified obstetricians only where foetuses were ≥ 23 weeks of gestation. The agent used to procure asystole was either lignocaine or potassium chloride (KCl). Oral mifepristone was administered, followed by misoprostol 24–48 h later to induce labour and delivery in all cases. Foetuses were then kept in cool storage within the mortuary at 4 °C until the time of PMMR.

### Image analysis

All radiologists were provided with the same clinical information: estimated date of delivery (or death), gestational age of fetus, prenatal ultrasound findings and clinical indication for iuMR or PMMR examination. None of the radiologists had access to the prenatal ultrasound images or autopsy results. A structured reporting template for recording the intracranial anatomical abnormalities and overall diagnosis was completed for all MRI examinations (see online electronic supplementary material).

The iuMR examinations were independently reported by a single radiologist with 15 years of experience in foetal imaging and without knowledge of the PMMR results. The PMMR studies were reported by two paediatric radiologists in consensus with 12 and 3 years post-mortem imaging experience, blinded to the iuMR results. The radiologists interpreting the PMMR were not involved in providing the primary clinical report for the PMMR. The radiologist interpreting the iuMRs was not involved in providing the original clinical interpretation for iuMR studies performed at sites 2 and 3, only at site 1.

### Autopsy and genetic testing

An autopsy was defined as any examination by a foetal pathologist, regardless of whether this was limited to external review, examination of the unfixed brain or organ retention with histopathological sectioning, dictated by parental consent. The pathologists were blinded to the PMMR interpretations undertaken for this study but had access to the iuMR reports. Genetic testing was performed at the discretion of foetal medicine specialists and pathologists in selected cases.

### Statistical analysis

Our primary outcome was level of concordance for final diagnosis of foetal brain malformation on iuMR and PMMR, with foetal autopsy where available as our reference standard. Descriptive statistics were performed and the case results collated in Microsoft Excel (Microsoft, Seattle, USA).

## Results

### Study cohort

Thirteen women and their singleton foetuses met our inclusion criteria with nine, two and two cases from institution sites 1, 2 and 3, respectively. The prenatal ultrasound findings and thus referral indications for further MRI imaging with gestational age of fetus are provided in Table 3.

**Table 3:** Individual case diagnoses and referral indications for foetuses included in this study

GA	Prenatal ultrasound findings	Intracardiac injection (ToP)	Intra-uterine foetal MRI diagnosis	PMMR diagnosis	Autopsy diagnosis	Comments	Genetic testing
Concordant cases							
29	ACC	KCl	ACC Left frontal cortical sulcation anomaly	ACC Left frontal cortical sulcation anomaly	ACC Left frontal cortical sulcation anomaly	All three investigations concordant (Fig. 1)	Normal microarray
22	Enlarged posterior fossa	None	DWM	DWM	DWM	All three investigations concordant	Normal microarray
Partial concordance							
22	Severe VM Small cerebellum TGA Vertebral anomalies	None	VM, aqueductal stenosis RES	VM Vertebral anomalies	VACTERL Vertebral and cardiac defects RES	Both imaging concordant for VM iuMR and PMMR discordant for RES and vertebral anomalies The autopsy confirms RES and vertebral anomalies with additional cardiac defects (Fig. 2)	None
23	ACC VM Microcephaly Small cerebellum No vermis	Lignocaine	ACC Enlarged ganglionic eminences Delayed sulcation PVNH Possible tubulinopathy or dystroglycanopathy	ACC, VM	Lissencephaly spectrum Technically limited due to maceration	iuMR and PMMR concordant for ACC and VM, although iuMR demonstrated additional PVNH Brain autopsy limited by maceration, lissencephaly spectrum diagnosed, ACC, and VM not able to detect	WES–TUBB1 pathogenic mutation (tubulin gene)
22	VM Small cerebellum Microphthalmia	None	Bilateral VM Small cerebellum Microphthalmia Possible aqueduct stenosis	Bilateral VM Small cerebellum. Microphthalmia ACC	Small cerebellum Microphthalmia	Imaging concordant for VM, small cerebellum, and microphthalmia Imaging discordant for aqueduct stenosis and ACC Autopsy did not identify the ACC or aqueductal stenosis	Array CGH = gain of chromosome 2q33.1, 0.24 Mb in size Variant of unknown clinical significance
22	Partial ACC Interhemispheric cyst	None	Hypogenesis CC Persistent BPC	Hypogenesis CC	Hypogenesis CC	iuMR and PMMR concordant for hypogenesis of CC but discordant for BPC Autopsy agrees with PMMR findings	Normal microarray
31	Severe VM	Lignocaine	ACC Abnormal bilateral cortical sulcation	ACC	ACC	iuMR and PMMR concordant for ACC but not cortical malformation Autopsy confirms PMMR findings	None
19	Posterior fossa cyst	None	Hypogenesis CC DWM	ACC DWM	No consent for brain autopsy	Imaging concordant for DWM, discordant for ACC No consent for brain autopsy (Fig. 4)	None
Discordant cases							
30	Bilateral VM Bilateral fixed flexion thumb deformity	None Intrapartum cephalocentesis performed	Severe VM Small dysmorphic cerebellum Aqueductal stenosis Suspected L1 CAM mutation	Large extra-axial haematomas Ventricles disrupted and collapsed	Extensive ventricular and extra-axial haematoma Collapsed ventricles Aqueductal stenosis Cerebellar heterotopias Absent medullary pyramids on histology	iuMR and PMMR discordant Autopsy identified many anomalies suspected on iuMR, and genetic testing confirmed the suspicions Intrapartum cephalocentesis hampered PMMR interpretation (Fig. 5)	Single-gene L1CAM mutation
23	Posterior fossa abnormality Small cerebellum	Lignocaine	Molar tooth malformation, absent cerebellar vermis	Normal	Absent cerebellar vermis	iuMR and PMMR discordant for absent cerebellar vermis Autopsy confirms iuMR findings (Fig. 6)	None
29	Bilateral VM	Lignocaine + KCl	MPPH syndrome	Normal	Postaxial polydactyly Non-diagnostic brain autopsy	iuMR and PMMR discordant Autopsy non-diagnostic Genetic testing confirms iuMR suspicions (Fig. 7)	WES—pathogenic PI3K mutation consistent with diagnosis of MPPH
28	VM	KCl	Unilateral intraventricular haemorrhage Periventricular venous haemorrhagic infarction	Normal	Non-diagnostic brain autopsy	iuMR and PMMR discordant Autopsy non-diagnostic	None
24	Severe VM	Lignocaine	Bilateral VM Subependymal nodular heterotopia Filamin A mutation suggested	Unilateral VM ACC	No consent for brain autopsy	iuMR and PMMR discordant for nodular heterotopia and ACC No consent for brain autopsy	FLNA mutation and maternal cranial imaging negative

*ACC* absent corpus callosum, *BPC* Blake’s pouch cyst, *CC* corpus callosum, *CSP* cavum septum pellucidum, *FLNA* filamin A, *iuMR* intra-uterine foetal MR, *KCl* potassium chloride, *L1CAM* L1 cell adhesion molecule, *MPPH* megalencephaly, perisylvian polymicrogyria, polydactyly and hydrocephalus, *PMG* polymicrogyria, *PMMR* perinatal postmortem MRI, *RES* rhombencephalosynapsis, *TGA* transposition of the great arteries, *ToP* termination of pregnancy, *VACTERL* vertebral, anal, cardiac, tracheoesophageal, renal and limb anomalies, *VM* ventriculomegaly, *WES* whole exome sequencing

The median gestational age at termination of pregnancy was 24.6 weeks with an interquartile range (IQR) of 22 to 29 weeks. The iuMR preceded the termination of pregnancy by less than 6 days in all cases. Median time between termination of pregnancy and post-mortem MRI was 133 h (i.e. 5.5 days; IQR 101–165 h, 4.2–6.9 days).

Termination of pregnancy was achieved via intracardiac lignocaine (*n* = 4), potassium chloride (*n* = 2), both lignocaine and potassium chloride together (*n* = 1) or no intracardiac injections (*n* = 6). In this latter subgroup, one foetal death occurred during Caesarean section where intrapartum cephalocentesis was performed due to severe cerebral ventricular dilatation. In the remaining five foetuses, only oral mifepristone and misoprostol were administered to the mother to induce delivery.

### Autopsy and genetic testing

Pathological assessment of the brain at autopsy was possible in 11 of the 13 cases. Of these, two cases were non-diagnostic and one was technically limited due to maceration-related changes.

Genetic testing was performed in eight (8/11, 72.7%) cases. A normal microarray was found in three cases. One case demonstrated an abnormality of unknown significance (gain of chromosome 2q33.1, 0.24 Mb in size). One test was negative for suspected filamin A mutation and three cases were positive for a specific abnormality (one PI3K mutation, one L1 cell adhesion molecule (L1CAM) mutation and one TUBB1 mutation).

### Foetal brain malformation diagnoses

Details of the iuMR, PMMR and autopsy findings for each case are outlined in Table 3. There were two cases where iuMR and PMMR were concordant; six cases where they were partially concordant and five cases for complete discordance.

#### Concordant cases

**Fig. 1:**
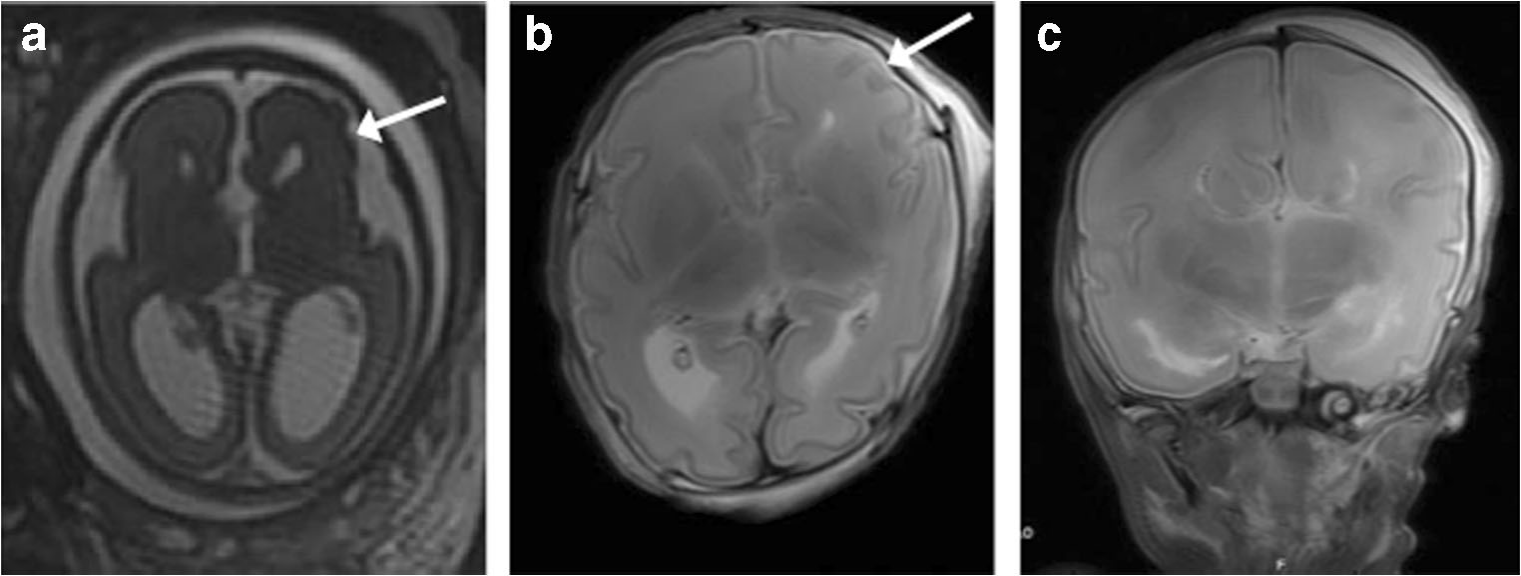
Agenesis of the corpus callosum (ACC) with unilateral left frontal oversulcation in a 29-week gestation fetus. **a** Axial T2-weighted imaging of the foetal brain on iuMR demonstrates the lack of callosal tissue and oversulcation in the left frontal lobe (white arrow). **b** Axial T2-weighted PMMR in the same fetus shows similar results, although the oversulcation (white arrow) is less marked. **c** Coronal T2-weighted PMMR of the fetus shows the typical steer-horn appearance in absent corpus callosum

Both iuMR and PMMR findings were concordant in two cases (2/13; 15.3%), one of Dandy Walker malformation and the other with complete callosal agenesis and unilateral frontal oversulcation (Fig. 1). The autopsy results were also concordant with both cases.

#### Partially concordant cases

There was partial concordance in six cases (6/13, 46.2%), of which five had autopsy correlation.

**Fig. 2:**
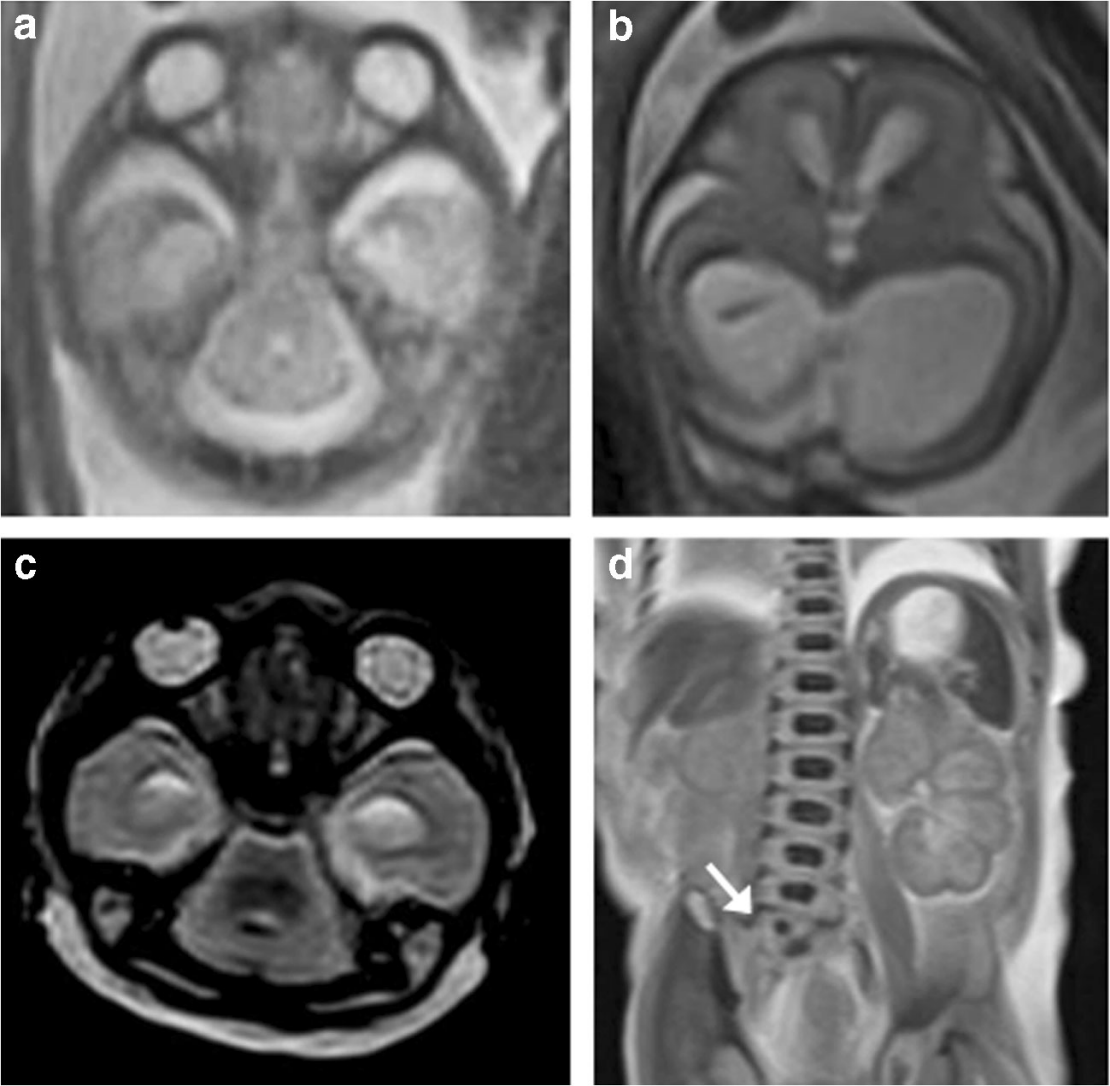
VACTERL spectrum anomalies in a 22-week gestation fetus. **a**, **b** Axial T2-weighted iuMR images at two different levels demonstrate a typical ‘ball’-shaped appearance of rhombencephalosynapsis (RES) and also lateral and third ventricular dilatation suggestive of aqueductal stenosis. **c** The T2-weighted PMMR image also shows a deficient cerebellar vermis with hemispheric fusion, although this was not appreciated. **d** Coronal T2-weighted PMMR of the body did however demonstrate a vertebral segmental anomaly (white arrow) that was not seen on iuMR

**Fig. 3:**
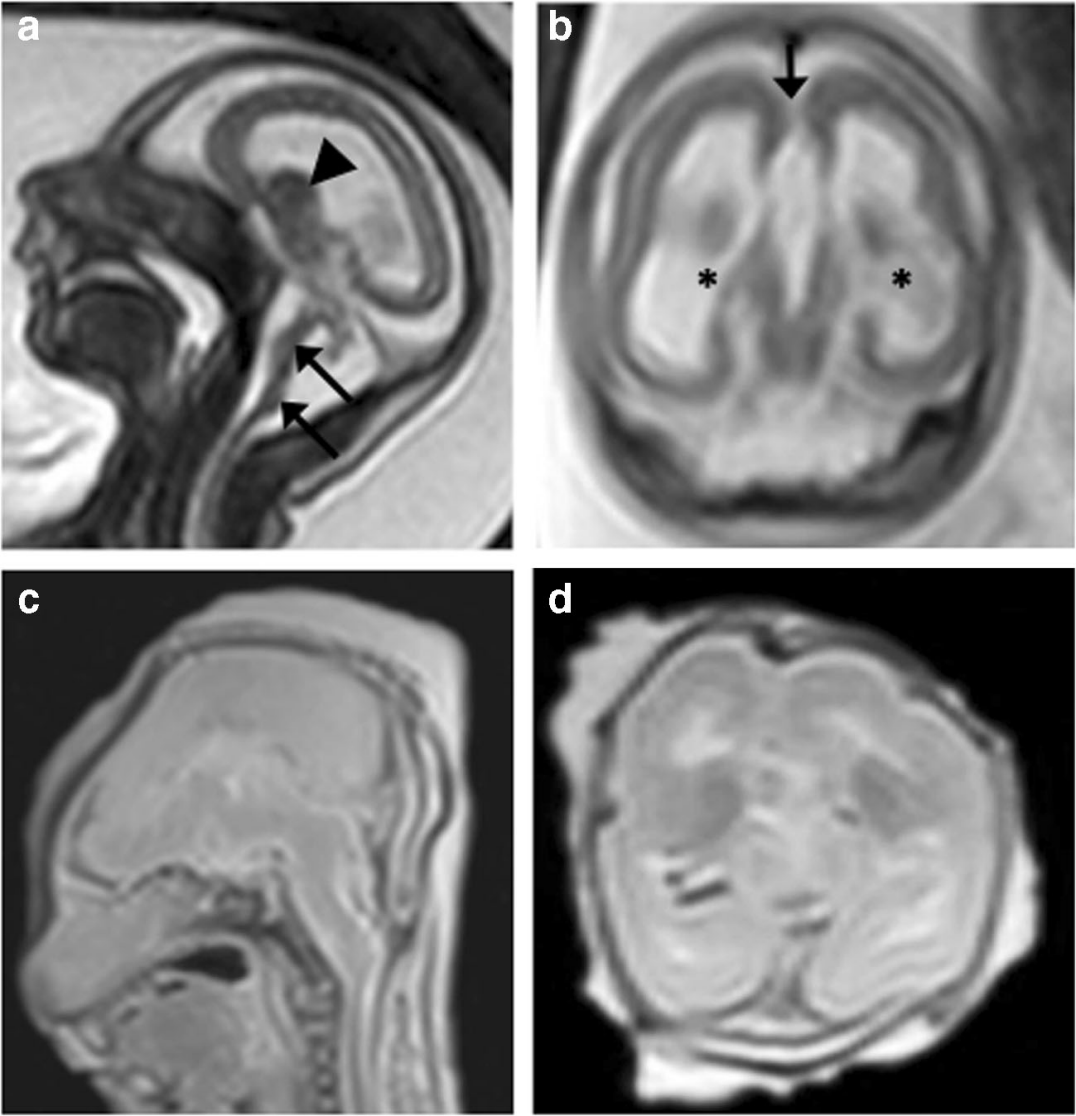
Tubulinopathy in a 23-week gestation fetus. **a** Axial T2-weighted iuMR demonstrates microcephaly, severely thinned hemispheric parenchyma, thinly kinked brainstem (black arrows), small malformed cerebellum with enlarged ganglionic eminence (arrowhead). **b** Coronal single shot T2-weighted iuMR image demonstrates bilateral lateral ventriculomegaly (asterisks) and an absent corpus callosum (black arrow). **c**, **d** Coronal and axial T2-weighted PMMR images show decompressed lateral ventricles. There is also difficulty in appreciating the intra-uterine cerebellar abnormalities

Of these five cases, PMMR ‘overcalled’ ventriculomegaly and ACC in one case, missed a rhomboencephalosynapsis (RES) in a case with vertebral, anal, cardiac, tracheoesophageal, renal and limb anomalies (VACTERL) (Fig. 2) and neuronal migration anomaly in a third case (Fig. 3). In the other two cases, the PMMR agreed with the autopsy findings. The iuMR ‘overcalled’ a Blake’s pouch cyst (BPC) for one case, abnormal bilateral cortical sulcation in another case and did not identify the vertebral anomalies in the case with VACTERL (Fig. 2).

**Fig. 4:**
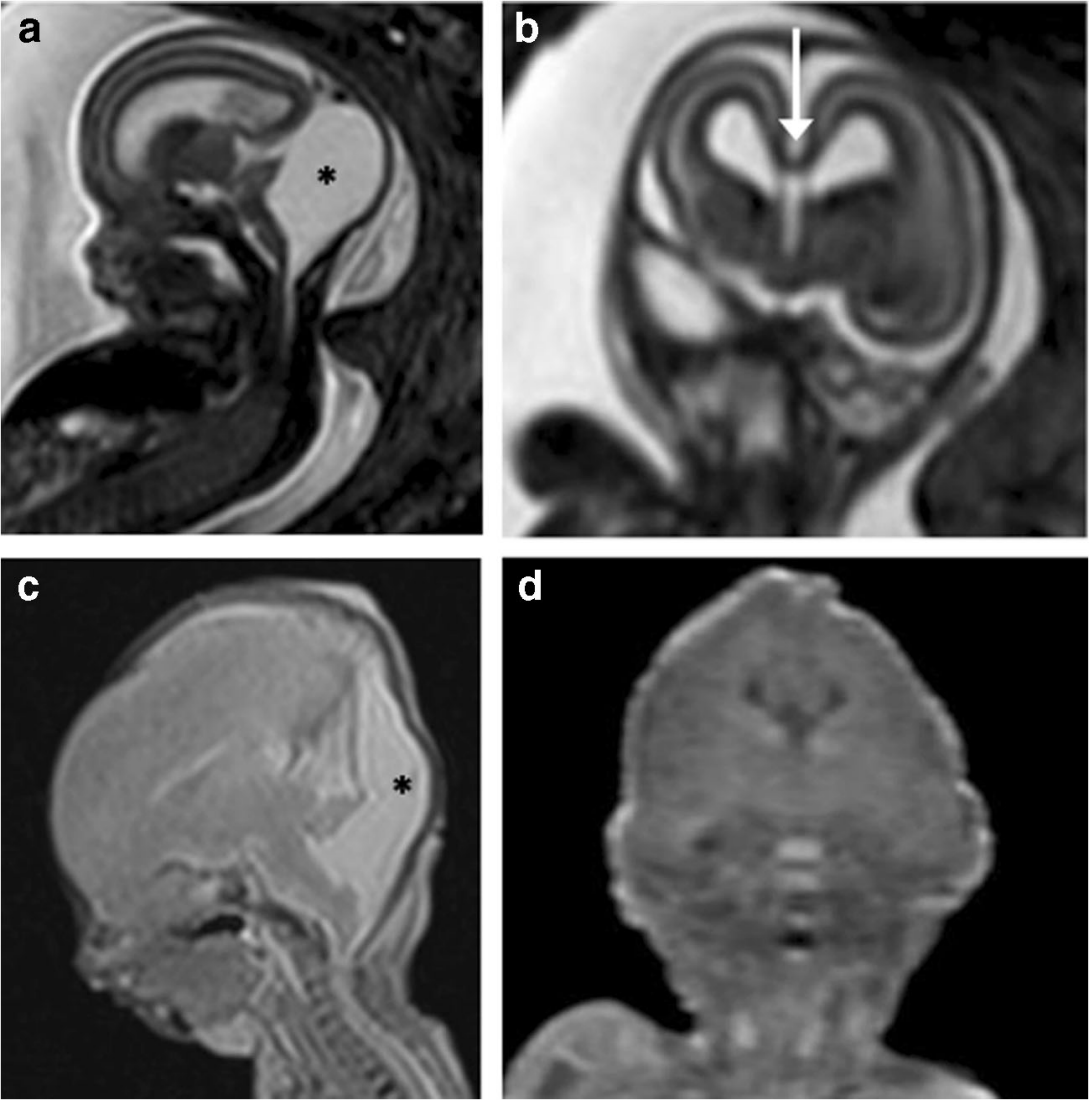
Dandy−Walker malformation (DWM) with callosal agenesis in a 19-week gestation fetus. **a** Sagittal iuMR showing upwardly rotated cerebellar vermis with large retrocerebellar CSF space (asterisk) enlarging the posterior fossa, consistent with an enlarged Blake’s pouch cyst. **b** Coronal T2-weighted iuMR demonstrates a thinned corpus callosum (white arrow) at the base of the anterior hemispheric fissure. **c** Sagittal T2-weighted and **d** coronal T1-weighted PMMR images were interpreted as a Dandy–Walker malformation with agenesis of the corpus callosum given the ‘steer-horn’ appearances of the lateral ventricles on the coronal view and posterior fossa cyst (asterisk)

In the one case where there was no autopsy correlation, both imaging modalities described a DWM but could not agree where there was absence or hypoplasia of the corpus callosum (Fig. 4).

#### Discordant cases

In the remainder of the cases (5/13, 38.5%), the findings between PMMR and iuMR were discordant. Of these, autopsy correlation was available for two cases. There was no parental consent for autopsy in one case, and in two cases, the autopsy was non-diagnostic.

**Fig. 5:**
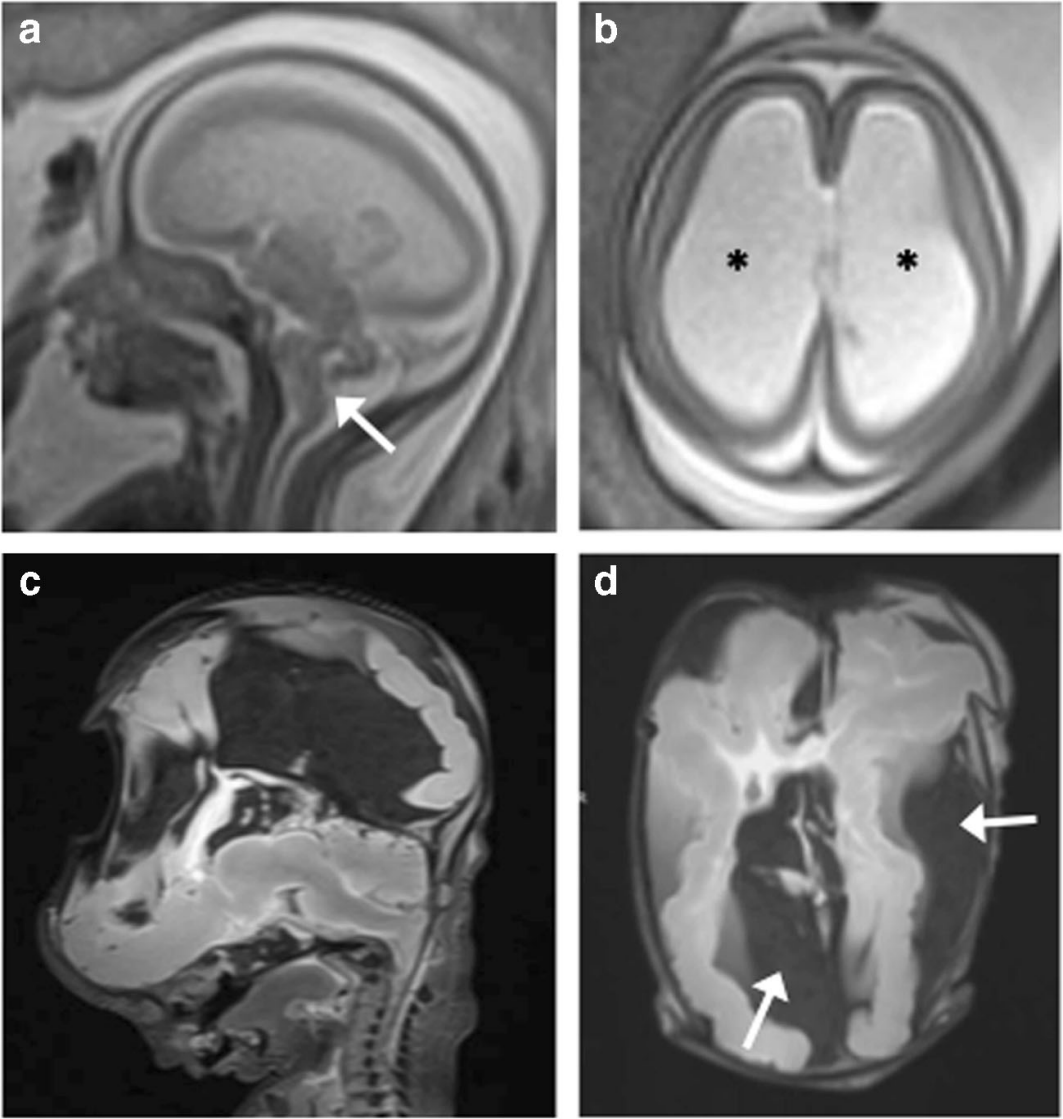
L1CAM (cell adhesion molecule) abnormality in a 30-week gestation fetus, with aqueductal stenosis. The fetus was also reported to have adducted thumbs on prenatal ultrasound examination. **a** Coronal and **b** axial T2-weighted iuMR images demonstrate kinking of the pontomesencephalic junction (white arrow), with severe ventriculomegaly (asterisks). The ganglionic eminences were not enlarged. **c** Sagittal and **d** axial T2-weighted PMMR following intrapartum cephalocentesis revealed collapsed ventricles and large extra-axial haematomas (white arrows). The haematomas were sustained during delivery but misinterpreted at PMMR as having been sustained prenatally

**Fig. 6:**
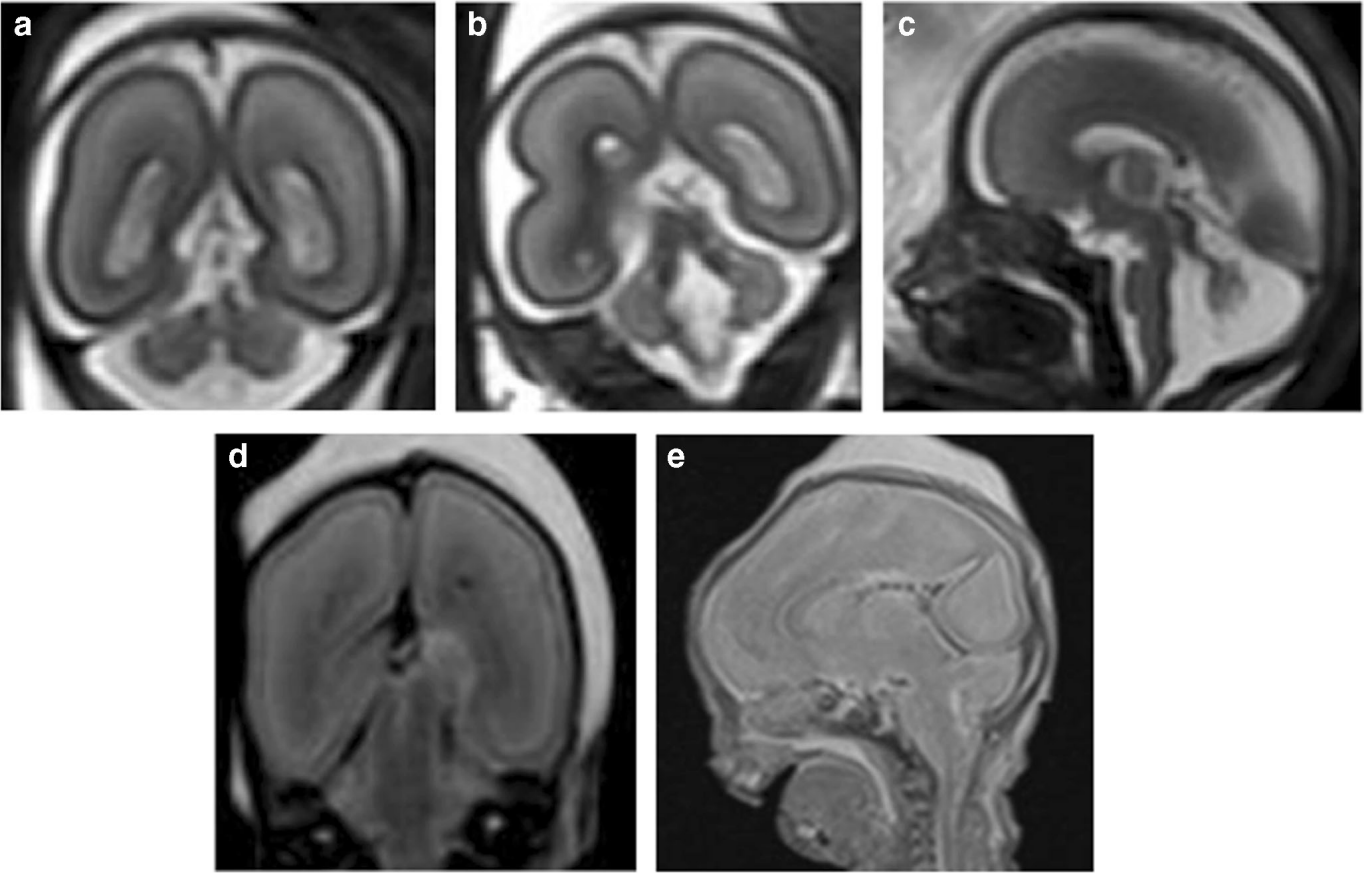
Abnormal cerebellar features in a 23-week gestation fetus. **a**, **b** Coronal T2-weighted iuMR images reveal features suggestive of an absent cerebellar vermis. The ‘buttocks sign’ is show in image **a**, and in image **b**, the ‘molar tooth appearance’ of elongated superior cerebellar peduncles is featured. **c** Sagittal T2-weighted iuMR image depicts the characteristic ‘figure of 7’ appearance of the elongated superior cerebellar peduncles and small cerebellum. **d**, **e** Coronal and sagittal T2-weighted PMMR images do not demonstrate the cerebellar abnormalities

In the two cases with autopsy reference, the PMMR did not identify any features of the final intracranial pathology. This included one case of suspected L1CAM mutation where the iuMR was able to demonstrate multiple findings (i.e. ventriculomegaly, dysmorphic cerebellum, kinked brainstem, aqueductal stenosis). However, given that this fetus had required cephalocentesis to allow delivery of the head via caesarean section, PMMR was only able to show extensive intracranial haemorrhage (Fig. 5). The other case was of a 23-week gestation fetus with an absent cerebellar vermis on iuMR, where the autopsy confirmed underlying Joubert’s syndrome (Fig. 6).

**Fig. 7:**
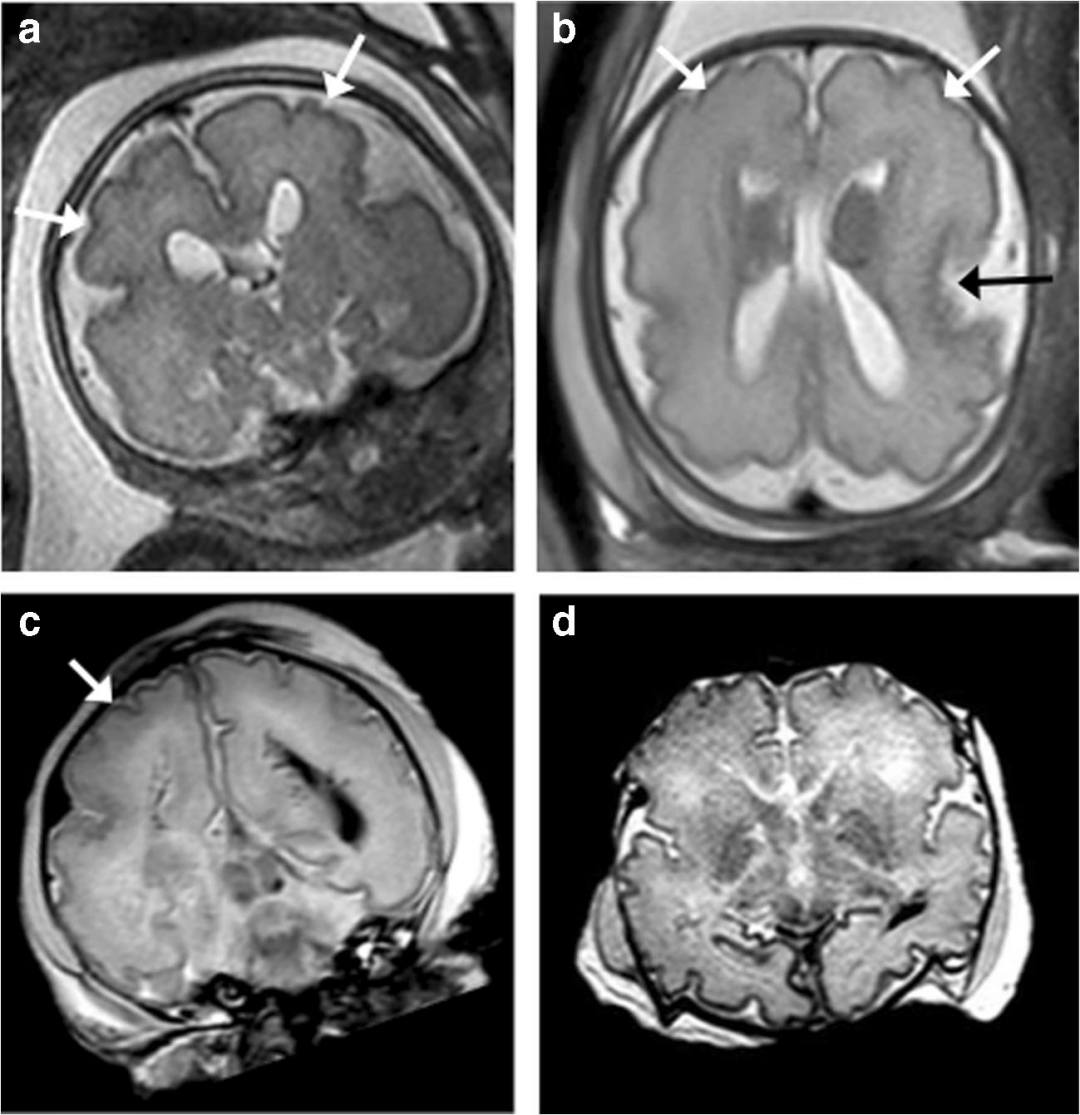
Megalencephaly polymicrogyria polydactyly and hydrocephalus (MPPH) syndrome due to PIK3R2 mutation in a 29-week gestation fetus. **a** Coronal and **b** axial T2-weighted iuMR images demonstrate bilaterally enlarged cerebral hemispheres and perisylvian polymicrogyria (white arrows). There was also a misshapen Sylvian fissure (black arrow). **c** Coronal and **d** axial T2-weighted PMMR images also demonstrate polymicrogyria (white arrows), particularly of the right cerebral hemisphere although this was harder to appreciate than on iuMR

In the two non-diagnostic autopsy cases, the PMMR was reported as normal; however, in one of these cases, the iuMR reported features of MPPH syndrome, which was later confirmed from genetic whole genome sequencing results (Fig. 7) and in the other iuMR demonstrated intraventricular haemorrhage and periventricular venous infarction. There was no genetic testing in this latter case.

In the final case where brain autopsy was declined, the PMMR depicted an absent corpus callosum and unilateral ventriculomegaly. The iuMR demonstrated bilateral ventriculomegaly with subependymal nodular heterotopia. A potential diagnosis for filamin A mutation was suggested but subsequently the genetic testing was found to be negative.

## Discussion

This study showed poor complete concordance between iuMR and PMMR for foetal brain malformations, particularly where conditions relied on a constellation of individual findings. Given that accurate phenotyping often facilitates specific genetic diagnoses, we interpret these results to mean that information on recurrence risk may be missed if PMMR examination is performed in the absence of iuMR, particularly after feticide has been performed. This has implications for parental genetic testing, pre-implantation genetic testing of embryos and tertiary referral for neurosonography and foetal MR for subsequent pregnancies.

The only other study in the literature comparable to our work was published by Izzo et al. [34], supporting similar conclusions. The authors reported that iuMR was able to demonstrate 79% of foetal brain abnormalities, compared with 45% by PMMR, using the combined results from iuMR and PMMR as a reference standard. For both modalities, the discrepancies were mostly due to false negative (i.e. ‘missed’) findings. At PMMR, these were more commonly due to CSF spaces, posterior fossa and brainstem-related findings as well as microcephaly and macrocephaly. For iuMR, the discrepancies were mainly related to lamination abnormalities or haemorrhagic lesions. Both modalities were reported to be similarly effective at identifying midline malformations and abnormalities of the ganglionic eminences.

In our study, the discrepant findings were due to both under- and overdiagnosis of callosal agenesis and underdiagnosis of other abnormalities that were key to diagnostic specificity, phenotyping and counselling about recurrence risk. These included brainstem and cerebellar malformations with specific pathological correlates (absent cerebellar vermis, kinking of the brainstem in L1CAM and tubulinopathy), perisylvian polymicrogyria, subependymal heterotopias and enlarged ganglionic eminences. It is interesting to note that only 20% (1/5) of the discordant cases did not have an intracardiac injection for termination of pregnancy, compared with 65% (4/6) partially concordant and 50% (1/2) concordant cases. In the case of the one discordant case that did not have intracardiac injection, there was intrapartum cephalocentesis performed during the delivery. These discrepant findings, although not statistically significant given our small numbers, seem to suggest that foetal intervention plays a part in the level of disagreement between the iuMR and PMMR findings. Feticide may increase foetal cerebral oedema and tissue lysis and mask some of the more subtle intracranial post-mortem findings. It may be possible that PMMR findings would show better concordance with iuMR and eventual autopsy in cases where feticide was not carried out, such as in stillbirths, intra-uterine deaths or cases of slightly lower gestational ages that do not require intracardiac injection for termination of pregnancy.

Whilst we demonstrated a greater disparity in iuMR and PMMR results, it is important to note several differences between our two studies. Firstly, the iuMR studies performed by Izzo et al. [34] were conducted on a 1.5-T magnet, rather than 3-T magnet in our case, and secondly over half of our foetuses (compared to none in the Izzo et al. study [34]) underwent termination of pregnancy with intracardiac potassium chloride injection. Finally, our case cohort were of a slightly more advanced gestational age which may have allowed certain anomalies to be better appreciated on iuMR, such as delayed sulcation and abnormalities of the Sylvian fissure. We also used independent radiologist reporting for iuMR and PMMR and deliberately used genetic testing and conventional PM (sometimes referred to as ‘genetic post-mortem’), where available, as the reference standard.

Our results contrast to the work by Whitby et al. [35], where they assessed the diagnostic accuracy of prenatal ultrasound and iuMR for foetal neurological anomalies, using the combined autopsy and PMMR examination as the reference standard. They found that in their 12 study subjects, iuMR provided a correct diagnosis for all cases, demonstrating a high level of concordance with PMMR and autopsy findings. This is unsurprising, as the accuracy or yield of PMMR was not assessed independently of the autopsy findings.

To our knowledge, there are no other studies that have assessed diagnostic accuracy rates for iuMR performed at 3 T and PMMR in the same cohort of patients, although several larger trials have assessed the accuracy for PMMR and iuMR individually, with autopsy or postnatal imaging as the reference standards.

For the detection of brain malformations on PMMR, an overall concordance rate of 73.8% (sensitivity 79.0%, specificity 71.7%) was reported for the detection of brain malformations on PMMR in an unselected population of 218 foetuses within the Magnetic Resonance Imaging Autopsy Study (MARIAS trial) [31] and Ashwin et al. [33] also found a high concordance rates of 92.2% (sensitivity 94.0%, specificity 90.9%) in a different cohort of 166 unselected foetuses. In these studies, the majority of the discrepancies were false positive findings, relating to intracranial haemorrhage, neuronal migrational and callosal anomalies. The true positive findings were usually due to a single intracranial abnormality rather than a combination of findings, and no specific details on genetic testing or unifying or syndromal diagnoses were provided.

These studies likely reflect the accuracy of PMMR for major rather than minor intracranial abnormalities, particularly as foetal neuropathology is very challenging even for the foetal pathologist, and autopsy may have been difficult to perform on some foetuses at early gestation. We do not challenge the use of PMMR as an excellent screening tool in unselected cases, but where significant neuropathology is identified on antenatal imaging our experience from this study showed that there were abnormalities which could not be seen on PMMR that were indisputably present in utero, confirmed at genetic post-mortem, and that would have been ‘missed’ if PMMR was the only imaging performed. The reasons for these discrepancies may have included anatomical distortion from intrapartum trauma [36], post-mortem cerebral oedema, collapse of cystic structures [37], descent and compression of posterior fossa structures or lack of recognition of severe macro- or microcephaly as diagnostic clues, due to the absence of normative data for post-mortem foetal biometry. There is also the possibility that foetal brain appearances may be less apparent on MR imaging following the iatrogenic effects from agents used to produce feticide [38]. Finally, whilst our post-mortem interval (time between delivery/death to imaging) was broadly consistent with other studies showing high PMMR concordance rates [31, 33] (i.e. less than 7 days), it has been suggested that a lower diagnostic yield may be seen when foetuses are imaged after a prolonged intra-uterine retention rate and later than 24 h from delivery [34]. However, this is challenging for foetuses greater than 24 weeks of gestation when intracardiac injection is routinely performed prior to onset of labour, and thus foetal demise precedes delivery by at least 36–48 h. For these reasons, the use of iuMR is required to ensure key diagnostic information is not ‘lost’ as a result of inevitable post-mortem changes [39].

The largest multicentre trial on iuMR diagnostic accuracy for foetal brain malformations (i.e. Magnetic Resonance Imaging to Enhance the Diagnosis of Fetal Developmental Brain Abnormalities In Utero (MERIDIAN) study) found a high concordance rate of 93% for iuMR [20] with autopsy and postnatal imaging and showed that iuMR was beneficial in aiding clinical management, regardless of whether the prenatal ultrasound findings are related to ventriculomegaly [40], commissural [41] and posterior fossa anomalies [42]. Nonetheless, there was a 7% error rate by iuMR (40/570 in the MERIDIAN cohort [43]), and imaging quality during iuMR can vary due to foetal motion artefacts, imaging technique and lack of isovolumetric images for multiplanar reconstruction of complex anatomy, due to the time-consuming nature of 3D acquisitions [44]. As such, post-mortem examinations will still be important for corroborating prenatal findings, and PMMR still has a place for families where invasive autopsies are refused [45, 46] as was the case in two of our subjects whose families permitted external PM only. In addition, in two other cases, post-mortem maceration adversely affected the ability of the pathologist to accurately perform a comprehensive autopsy.

Limitations of this study include the retrospective nature of the work and small sample size. To address our specific research question, there was a referral or selection bias, given that the MRI examinations were conducted for foetuses with suspicion of brain malformations based on tertiary prenatal US. In addition, because this was a retrospective study, our pathologists were not blinded to either the iuMR findings or the PMMR results. However, complete agreement of the iuMR diagnosis with the results of whole exome or specific genetic testing in three cases could not be influenced by unblinding of the pathologist.

The strengths of this study include the multicentre origin of the case load, iuMR performance at 3 T and the use of ‘expert’ readers in the independent interpretation of iuMR and PMMR, blinded to each other’s radiological opinions. We also include the autopsy data and genetic testing results, where available, as our reference standard against which the index tests were measured.

In summary, we advocate performing iuMR for accurate diagnosis of foetal brain malformations in cases where there is a known abnormality or suspicion based on tertiary prenatal ultrasound, independent of post-mortem imaging. iuMR can provide additional information for improved phenotyping of the brain malformations and thus a better chance of targeting and interpreting genetic testing. This is particularly important in the age of next-generation sequencing technology using whole exome sequencing (WES) [11, 47], as a single gene or panel testing may be more appropriate and cost-effective. Further work in a larger sample size with complete autopsy as a reference standard will enable a more robust evidence basis to inform future clinical practice.

## Conclusion

Where tertiary prenatal ultrasound detects or suggests a foetal brain abnormality, iuMR should be performed to improve foetal phenotyping and diagnostic accuracy. Reliance on PMMR alone may result in misdiagnosis and incorrect phenotyping in the majority of cases.

## Electronic supplementary material

ESM 1 (DOCX 13 kb)

## Abbreviations

ACC: Absent corpus callosum
BPC: Blake’s pouch cyst
CC: Corpus callosum
CSF: Cerebrospinal fluid
CSP: Cavum septum pellucidum
ETL: Echo train length
FLNA: Filamin A
FOV: Field of view
FSE: Fast spin echo
HASTE: Half-Fourier acquisition single shot turbo spin echo
iuMR: Intra-uterine magnetic resonance imaging
KCl: Potassium chloride
L1CAM: L1 cell adhesion molecule
MARIAS: Magnetic Resonance Imaging Autopsy Study
MERIDIAN: Magnetic Resonance Imaging to Enhance the Diagnosis of Fetal Developmental Brain Abnormalities In Utero
PMG: Polymicrogyria
MPPH: Megalencephaly, perisylvian, polymicrogyria, polydactyly and hydrocephalus
MRI: Magnetic resonance imaging
PMI: Post-mortem interval
PMMR: Post-mortem magnetic resonance imaging
RES: Rhombencephalosynapsis
SE: Spin echo
SPACE: Sampling perfection with application optimised contrasts using different flip angle evolution
SSFSE: Single shot fast spin echo
TE: Echo time
TGA: Transposition of the great arteries
TI: Inversion time
ToP: Termination of pregnancy
TR: Repetition time
TSE: Turbo spin echo
VACTERL: Vertebral, anal, cardiac, tracheoesophageal, renal and limb anomalies
VM: Ventriculomegaly
WES: Whole exome sequencing
