# Supplementary material for: Diagnostic assessment of foetal brain malformations with intra-uterine MRI versus perinatal post-mortem MRI
Source: Neuroradiology. 2019 May 10;61(8):921–34. doi: 10.1007/s00234-019-02218-9 (PMC6620257; doi:10.1007/s00234-019-02218-9)
Supplement: Supplementary file 1 — (DOCX 13 kb) [file 234_2019_2218_MOESM1_ESM.docx]

**Electronic Supplementary Material**

**Online Supplementary Figure 1**

Reporting Template for

**CLINICAL INDICATION: [<>]**

Fetal Head:

Calvarium: [<>]

Orbits: [<>]

Palate: [<>]

Posterior choanae: [<>]

Mandible: [<>]

If potential micrognathia, state inferior facial angle (<50 degrees abnormal)

External auditory canal, tympanic cavity, inner ear structures:

Tongue (protrusion or glossoptosis?): [<>]

Nasal bone: [<>]

Nuchal fold: [<>]

Globes / orbits: [<>]

Optic nn / chasm: [<>]

Pituitary gland / stalk: [<>]

Ventricular system:

Lateral [<>]

Third [<>]

Fourth [<>]

Extraaxial CSF spaces: [<>]

Hemispheres: [<>]

Septum pellucidum: [<>]

Corpus callosum: [<>]

Sulcation: [<>]

Cerebellum: [<>]

Vermis foliation [<>]

Tegmentovermian angle [<>]

Hemispheres [<>]

Retrocerebellar CSF space [<>]

Brainstem: [<>]

Diffusion weighted imaging: [<>]

Susceptibility weighted imaging: [<>]

**IMPRESSION AND RECOMMENDATIONS:**

[<>]
